# Cytocompatibility of Potential Bioactive Cerium-Doped Glasses based on 45S5

**DOI:** 10.3390/ma12040594

**Published:** 2019-02-16

**Authors:** Gianluca Malavasi, Roberta Salvatori, Alfonso Zambon, Gigliola Lusvardi, Luca Rigamonti, Luigi Chiarini, Alexandre Anesi

**Affiliations:** 1 Dipartimento di Scienze Chimiche e Geologiche, Università degli Studi di Modena e Reggio Emilia, via G. Campi 103, 41125 Modena, Italy; alfonso.zambon@unimore.it (A.Z.); gigliola.lusvardi@unimore.it (G.L.); luca.rigamonti@unimore.it (L.R.); 2 Laboratorio dei Biomateriali, Dipartimento di Scienze Mediche Chirurgiche Materno-Infantili e dell'Adulto, Università di Modena e Reggio Emilia, Via G. Campi 213/A, 41125 Modena, Italy; roberta.salvatori@unimore.it (R.S.); luigi.chiarini@unimore.it (L.C.); alexandre.anesi@unimore.it (A.A.)

**Keywords:** bioactive glasses, cerium oxide, hydroxyapatite, cytocompatibility, cell viability, cell proliferation

## Abstract

The cytocompatibility of potential bioactive cerium-containing (Ce^3+^/Ce^4+^) glasses is here investigated by preparing three different glasses with increasing amount of doping CeO_2_ (1.2, 3.6 and 5.3 mol% of CeO_2_, called BG_1.2, BG_3.6 and BG_5.3, respectively) based on 45S5 Bioglass® (called BG). These materials were characterized by Environmental Scanning Electron Microscopy (ESEM) and infrared spectroscopy (FTIR) after performing bioactivity tests in Dulbecco’s Modified Eagle Medium (DMEM) solution, and the ions released in solution were determined by Inductively Coupled Plasma Mass Spectrometry (ICP-MS) and Optical Emission Spectrometry (ICP-OES). The data obtained clearly show that the glass surfaces of BG, BG_1.2 and BG_3.6 were covered by hydroxyapatite (HA), while BG_5.3 favored the formation of a cerium phosphate crystal phase. The cytotoxicity tests were performed using both murine long bone osteocyte-like (MLO-Y4) and mouse embryonic fibroblast (NIH/3T3) cell lines. The cerium-containing bioactive glasses show an increment in cell viability with respect to BG, and at long times, no cell aggregation and deformation were observed. The proliferation of NIH/3T3 cells increased with the cerium content in the glasses; in particular, BG_3.6 and BG_5.3 showed a higher proliferation of cells than the negative control. These results highlight and enforce the proposal of cerium-doped bioactive glasses as a new class of biomaterials for hard-tissue applications.

## 1. Introduction

In the last years, rare earth elements have received much attention in the biomaterials field due to their specific properties [1]. Among these elements, cerium presents very interesting features thanks to its ability to easily and drastically adjust its electronic configuration to best fit its surrounding environment [2]. In particular, cerium oxide nanoparticles (CeONPs or nanoceria) have received much attention because of their excellent catalytic activities, which are derived from quick interconversion between the oxidation states Ce^4+^ and Ce^3+^ [3]. This ability is due to a specific feature of the surface of CeONPs: it exhibits oxygen vacancies in the lattice structure arising from the loss of oxygen atoms, alternating between CeO_2_ and CeO_2−*x*_ during redox reactions [4,5]. According to Pirmohamed and Heckert, nanoceria has been also recently found to have multi-enzymatic mimetic properties in physiological environment, including superoxide dismutase (SOD), catalase (CAT) and oxidase (OXI) [6,7]. This produces various positive biological effects, such as antioxidant towards almost all noxious intracellular reactive oxygen species (ROS), which stoke the inflammation [8] after surgical operations, as well as for those involving implantation of biomaterials, the so-called surgical stress response [9,10]. For these reasons, nanoceria has emerged as a material in biological fields such as bioanalysis, biomedicine, drug delivery, and bioscaffolding [11].

Among biomaterials for implantology, bioactive glasses are a class of materials widely used for their ability to form chemical bonds with soft and/or hard body tissues (bones and teeth) [12], feature known as bioactivity [13]. In particular, materials that show in vitro formation of hydroxyapatite (HA) on their surface when tested in simulated body fluid (SBF) solutions have been demonstrated to show also in vivo bioactivity [14]. Indeed, the formation of an HA layer on the surface of the implanted samples permits an optimal bond with the bones [15].

Since the discovery of the first bioactive glass, 45S5 Bioglass® (hereafter called **BG**) [13], the research activity has been focused to improve its properties as biomaterial by modifying the original composition: 45% SiO_2_, 24.5% Na_2_O, 24.5% CaO and 6% P_2_O_5_ in %weight, corresponding to a molar composition (mol%) of SiO_2_ 46.1%, Na_2_O 24.4%, CaO 26.9%, and P_2_O_5_ 2.6% [16]. In particular, the **BG** composition has been modified by addition of other oxides whose constituents (i.e., metallic ions) can produce specific effects in the biological environment after their physiological release [17,18]. For example, addition of magnesium or strontium to the glass matrix helps bone formation [19,20], while zinc enhances the recovery from inflammation in addition to bone growth [21]. Furthermore, the introduction of specific species on the bioactive glass surface interacting with the physiological environment could promote important features such as the bacteriostatic activity [22,23,24].

In this context, potential bioactive glasses based on modification of **BG** composition with Ce^4+^/Ce^3+^ (addition of CeO_2_ in the batch during the glass synthesis) were developed in the past years in order to unify the ability of the material to promote the binding with hard tissues (bioactivity, HA formation) with simultaneously enzymatic-like activities (CAT and SOD). In fact, Nicolini et al. [25,26,27] have shown how glasses with BG composition modified with up to 5.3 mol% of CeO_2_ present both CAT- and SOD-mimicking abilities, as also non-stoichiometric CeONPs do. In particular, CAT-like activity is dependent on the content of dopant, and it increases with the increase of cerium content. Moreover, the bioactivity in terms of HA formation during tests in SBF decreases as a function of CeO_2_ quantity. Although SBF tests are usually firstly applied to determine the bioactivity of a material, the results should be also interpreted carefully. In fact, in recent years the reliability of SBF tests has been often criticized, and several researchers have pointed out that the apatite-forming ability in SBF, i.e., the formation of an HA layer on the material, cannot be assumed as a direct prediction of in vivo bioactivity [28]. In particular, SBF contains only inorganic ions with concentrations similar to those of human plasma, and therefore the assumption to mimic the complex physiological environment looks simplistic. On the other hand, in vitro cell culture assays are nowadays quite rapid and standardized, and they are fundamental in order to determine the biocompatibility of new materials intended for biomedical applications [29]. In particular, such tests are essential to develop new biomedical materials for their application (repair and replace diseased or damaged bones and as scaffold) [30,31] and to modulate their cell viability and proliferation.

The potential in vivo applications of these materials needs their testing for cytocompatibility [32,33], especially after the results obtained on cerium-doped bioglasses in terms of enzymatic-like CAT and SOD activities [25,27]. Therefore, in this paper three glasses based on BG and modified with increasing amount of doping CeO_2_ (1.2, 3.6 and 5.3 mol% of CeO_2_, called BG_1.2, BG_3.6 and BG_5.3, respectively) have been synthesized and tested in cell culture medium for their cytocompatibility with murine long bone osteocyte-like (MLO-Y4) and mouse embryonic fibroblast (NIH/3T3) cell lines.

## 2. Materials and Methods

### 2.1. Synthesis of Glasses

Reagent-grade SiO_2_, Na_2_CO_3_, CaCO_3_, Na_3_PO_4_·12H_2_O and CeO_2_ in about total 100 g were mixed in an agate mortar in the desired ratio, and then melted in an electric oven into a platinum crucible (8853, Milan, Italy). The heating ramp was set to 15 °C/min from room temperature to 1000 °C and 8 °C/min from 1000 up to 1350 °C. The samples were maintained at 1350 °C for 2 h to ensure the optimal melting and mixing of all the oxides, and finally they were quenched at room temperature on a graphite mold in order to form a cylindrical bar of 1.1 cm in diameter. The bar was annealed at 400 °C for 2 h in order to reduce internal stress. The bar was then cut with a saw bearing a diamond blade in order to obtain discs of 1 mm of thickness and a surface of about 2 cm^2^. The compositions of the synthesized glasses are reported in Table 1.

### 2.2. In Vitro HA Formation Tests

In vitro HA formation ability of BG, BG_1.2, BG_3.6 and BG_5.3 was verified by soaking glass samples in cell culture medium employed for the cytocompatibility tests (see below). In particular, discs were soaked in Dulbecco’s Modified Eagle Medium (DMEM) (Invitrogen, Karlsruhe, Germany) at 37 °C for 5 days. Then the disks were separated from the extract by filtration using a 0.22-micron filter (Merck Millipore, Darmstadt, Germany). The extract was used for the indirect-contact cytotoxicity method, while the disks were analyzed in order to verify the HA formation on the glass surfaces as reported in the following paragraphs.

#### 2.2.1. Environmental Scanning Electron Microscopy–Energy Dispersive Spectroscopy (ESEM–EDS)

After soaking, images of the surfaces of the different glasses were collected in order to gain the morphological and elemental characterizations of the surfaces and to determine if there were detectable apatite-like areas. The morphological and elemental analyses were carried out by means of ESEM-EDS using a FEI Quanta 200 Instrument (Fei Company, Eindhoven, The Netherlands), equipped with an INCA 350 EDS apparatus (Oxford Instruments, Abingdon, UK); EDS analyses were performed over three different areas of the sample surface and the maximum value of standard deviation (SD) was 0.5%.

#### 2.2.2. Fourier Transform Infrared (FTIR) Spectroscopy

After soaking, glass surfaces were also scraped with a steel blade and the resulting powders analyzed with the infrared spectrometer FTIR 4000 (Jasco, Tokyo, Japan) in the 400–4000 cm^−1^ wavenumber range. Measurements were performed as KBr disks prepared mixing 1 mg of sample and 200 mg of KBr. Spectra were collected as mean of 64 scans and plotted as absorbance vs. wavenumbers (cm^−1^).

#### 2.2.3. Leaching of Silicon, Calcium, Sodium, Phosphorus and Cerium

Leaching tests were performed on different replicated samples in order to verify the amount of silicon, calcium, sodium, phosphorus and cerium released during the cellular tests. The obtained solutions were analyzed through Inductively Coupled Plasma Optical Emission Spectrometry (ICP-OES) for the determination of silicon, calcium, sodium and phosphorus with the Optima 5300 DV spectrometer (Perkin Elmer, Shelton, CT, USA), and through Inductively Coupled Plasma Mass Spectrometry (ICP-MS) for cerium by means of an HR-MC-ICPMS Neptune (Thermo Fisher Scientific Instrument, Bremen, Germany).

In particular, glass disks were soaked in DMEM with a glass surface area/DMEM volume ratio of 6 cm^2^/mL at different times (1 and 72 h) and 37 °C. The ratio between surface sample area and DMEM equal to 6 cm^2^/mL was chosen to apply the same conditions as the cellular tests proposed in the method ISO 10993-5 [34]. Then disks were separated from the DMEM extracted solution by filtration using a 0.22 micron filter (Merck Millipore, Darmstadt, Germany) in order to eliminate the possible glassy debris particles larger than 0.22 micron that could be formed during the tests. Each solution was analyzed and silicon, sodium, calcium, phosphorus and cerium concentrations are expressed as mean value with 5% of SD over the replicates.

### 2.3. Cytocompatibility Assays

MLO-Y4 and NIH/3T3 cell lines were cultured in DMEM (Euroclone, Milan, Italy) supplemented with 10% fetal bovine serum (Euroclone, Milan, Italy), 100 μg/mL pen-streptomycin (Invitrogen-Thermo Fisher Scientific Corporation, Whaltham, MA, USA) and sodium pyruvate 1 mM (Euroclone, Milan, Italy) at 37 °C in a humidified atmosphere of 5% CO_2_ in air, and used in the cytocompatibility experiments. All samples were employed in both direct (neutral red uptake) and indirect (extract) contacts for the cytocompatibility assays (XTT test and BrdU assays, see below).

#### 2.3.1. Neutral Red (NR) Uptake

NR uptake (NR solution N2889 Sigma-Merck, Darmstadt, Germany) is a sensitive and widely used assay to evaluate the number of viable cells in a culture [35]. It is based on the capability of viable cells to accumulate the supravital NR dye in their lysosomes. Cytotoxicity is calculated as a reduction of the NR uptake after 24, 48 and 72 h of chemical exposure to the direct material into the well. Cells were seeded in a six multiwell and cultured at 37 °C ± 1 °C, 90% ± 5% humidity, and 5 ± 1% CO_2_/air for 24, 48 and 72 h. 300 μL of NR solution were added after removing culture medium on the well for 3 h. NR solution was discarded and cells rinsed with 300 μL of Dulbecco’s-Phosphate Buffer Solution (D-PBS). Ethanol/acetic acid mixture was added in order to extract the dye from cells. Quantity of extracted NR was measured using UV-visible spectrophotometry at 540 nm (Multiscan RC by Thermolab, ThermoFisher Scientific, Helsinki, Finland). All experiments were repeated three times for each analyzed sample and using DMEM without serum (CTRL−) and phenol 0.45% (CTRL+) as references.

#### 2.3.2. XTT Test

XTT is a colorimetric assay for non-radioactive quantification of cell viability. Metabolic-active cells reduce the yellow tetrazolium salt XTT in an orange formazan dye, and this can be directly quantifiable by spectrophotometry; live cells have mitochondrial enzymes that perform this action [36]. 96 well-cultured plates were used to culture cells in contact with sample extract and incubated for 24, 48 and 72 h. Finally, XTT labelling solution (Cell Proliferation Kit II (XTT) Roche diagnostics, Indianapolis, IN, USA) was added (final concentration 0.3 mg/mL) in every well after 4 h. Colorimetric response due to the orange formazan dye solution was counted by measuring the absorbance at 490 nm, using the UV-visible spectrophotometer reported above. As before, DMEM without serum and phenol 0.45% were used as CTRL− and CTRL+, respectively. The sample extract was made in centrifuge tubes with a ratio between sample and extracting solution (DMEM without bovine fetal serum) equal to 6 cm^2^/mL (according to ISO 10993-5 [34]). Vials were incubated at 37 °C for 72 h and then pH was measured and adjusted to have physiological cell conditions. Every extract was filtered with a 0.22 micron filter before use.

#### 2.3.3. 5-Bromo-2-deoxyUridine (BrdU) Test

BrdU is an analogue of thymidine and it can be incorporated into the newly-synthetized DNA of cycling cells. Specific antibodies react with free BrdU and also with BrdU incorporated into DNA. Binding of the antibody requires denaturation of the DNA. Cell proliferation was evaluated by BrdU test, which is a colorimetric assay [37]. BrdU labelling solution was added in cells grown in 96-well plates, and after 24 h exposure to the samples extracts the proliferation was evaluated by absorbance at 370 nm, using the UV-visible spectrophotometer reported above. The amount of the detected signal is then correlated to the intensity of the newly synthesized DNA, and consequently to the number of proliferating cells in the culture.

#### 2.3.4. Morphological Evaluations

After every period of incubation (samples with cells) of the different tests reported in the previous paragraphs, the morphology of cells was evaluated using optical microscopy (Nikon TMF, Tokyo, Japan). MLO-Y4 cells were also suspended in 5 mL of cell growth medium and then placed on the glass disks. After incubation for 72 h, the samples were placed in an Eppendorf vessel and fixed overnight with 2.5% glutaraldehyde in sodium cacodylate 0.1 M buffer solution (pH = 7.2). Then the samples were washed with the buffer, fixed in 1% buffered osmium tetroxide for 1 h, dehydrated in a graded series of ethanol and embedded in Epon-araldite resin. Examination of the cell morphology was then performed using the ESEM-EDS instrument reported above.

#### 2.3.5. Statistical Analysis

One-way variance analysis (ANOVA) was used for statistical analysis of all results. Static test compares the means between the groups treated with samples and negative/positive references, and determines whether any of those means are statistically and significantly different from each other.

## 3. Results and Discussion

The modified glasses BG_1.2, BG_3.6 and BG_5.3 were prepared adding increasing amount of CeO_2_ to the starting composition of BG, rather than substituting one of the other oxides. This was done in order to avoid the alteration of the ratio between the original components SiO_2_, Na_2_O, CaO and P_2_O_5_ of the bioactive glass, which should ensure the desired bioactivity, while having CeO_2_ incorporated in the BG matrix.

**Figure 1:**
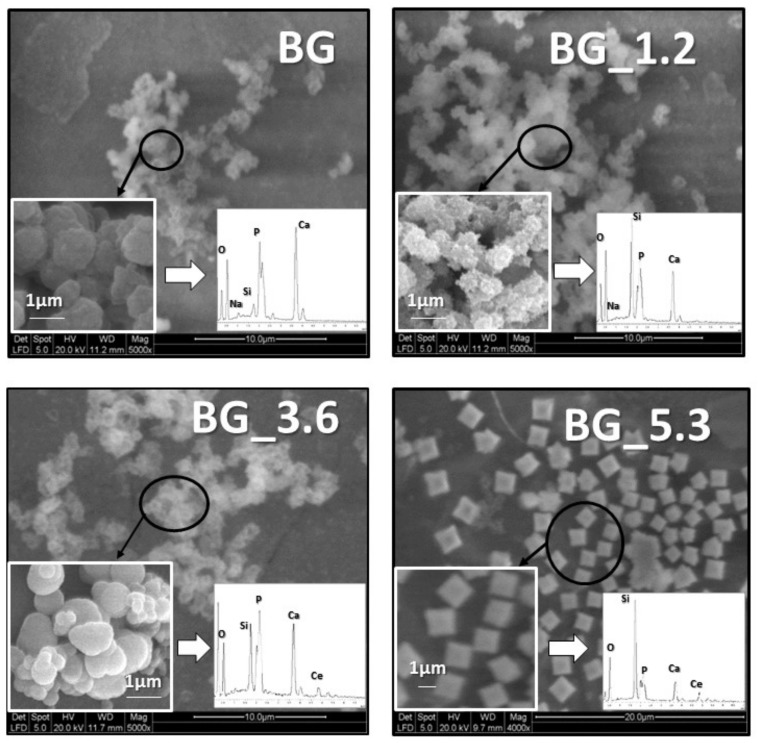
ESEM images of BG, BG_1.2, BG_3.6 and BG_5.3 surfaces after 5 days of soaking in DMEM at 37 °C. Insets: Images at higher magnification and EDS spectra with elemental assignment of the observed peaks.

The ESEM micrographs of the sample surfaces after being soaked in DMEM, together with the corresponding EDS analyses, are reported in Figure 1. As clearly noticeable, the glass surfaces were partially covered by new phases. In the case of BG, the morphology and the Ca/P ratio (1.70) are characteristic of HA (theoretical Ca/P ratio 1.67). For BG_1.2 and BG_3.6, the morphologies are similar to the one of BG but the Ca/P ratios slightly decrease to 1.48 and 1.50, respectively, showing a depletion of calcium. This can be probably ascribed to the presence of cerium, as detected by EDS spectrum in the phosphate-rich phase formed on the sample surface. The EDS analysis performed on BG_5.3 surface shows a further slight decrease in the Ca/P ratio (1.39) and a consequent increase in cerium, with considerable changes in the morphology. In fact, there are some more regular aggregates with very evident edges compared to the previous samples. Furthermore, in the EDS spectra shown in Figure 1, also Si (often very intense) and Na (slightly intense) signals can be observed. This is because the volume of matter investigated includes both the new formed particles on the material surface and the underneath glass surface.

**Figure 2:**
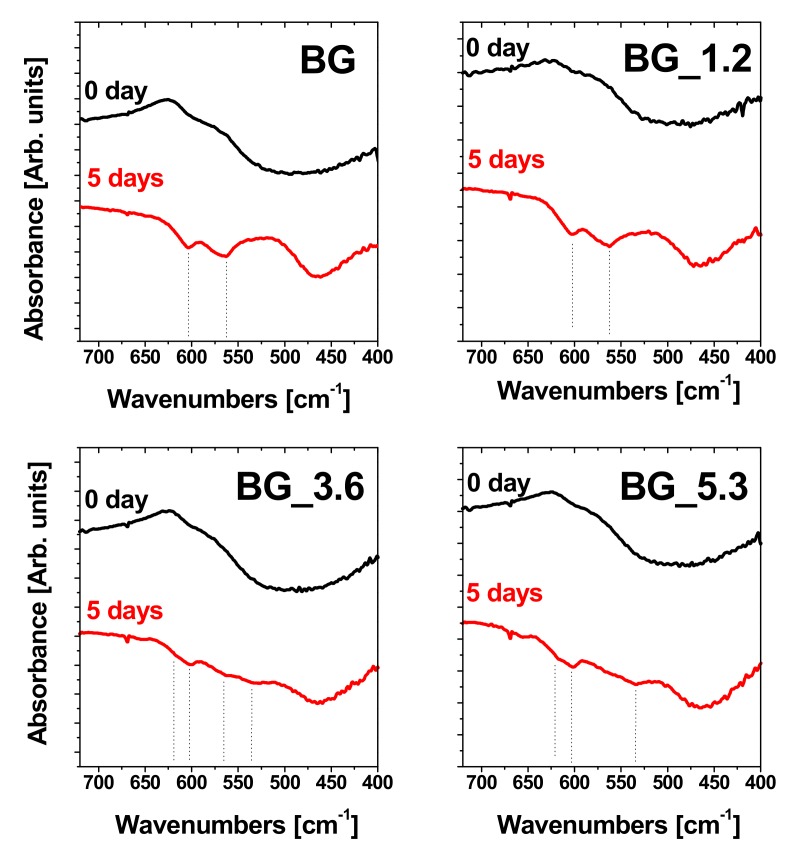
FTIR spectra in the 700–400 cm^−1^ spectral range of BG, BG_1.2, BG_3.6 and BG_5.3 before (0 day) and after (5 days) soaking in DMEM at 37 °C.

The FTIR spectra on glass samples before and after soaking in DMEM for 5 days are reported in Figure 2. This analysis was performed in the specific 700–400 cm^−1^ spectral range in order to verify the presence of the diagnostic bands of apatite-like crystalline phase at 605 and 565 cm^−1^ [38] after soaking the glasses in DMEM. In the 500–650 cm^−1^ region also the three signals at 620, 566 and 535 cm^−1^ due to the crystal phase of CePO_4_ are present [26]. In spite of this overlapping, the signals at 605 and 565 cm^−1^ of HA were detected for all the samples, although the intensity of the signals is higher for BG and BG_1.2. The bands attributable to CePO_4_ are clearly visible only in the spectrum of BG_5.3. This is correlated with the high cerium-content in the glass, which causes a high amount of cerium ions both on the glass surface and released in solution (see below). As a consequence, the high cerium concentration favors the formation of cerium phosphate on the surface to the detriment of HA, thus the bioactivity of the glasses decreases with the increasing content of cerium [39].

In Table 2 silicon, calcium, sodium, phosphorus and cerium concentrations in DMEM solution before (*t* = 0) and after the leaching tests are reported. The cerium is always lower than 1 ppm and dependent on the cerium-content of the glass matrix. This low concentration can be explained by the tendency of cerium ions released from the glass to form insoluble species (probably phosphate compounds), which precipitate on the glass surfaces. This is also confirmed by the low amount of phosphorous detected after the leaching tests for the glasses with higher cerium content (BG_3.6 and BG_5.3) with respect to the before concentration. The maximum amount of sodium released is detected for BG_1.2 in all conditions, suggesting that this glass should possess the lowest chemical durability. The same trend can be observed for the remaining elements silicon and calcium, for which the maximum release is detectable for BG_1.2.

**Figure 3:**
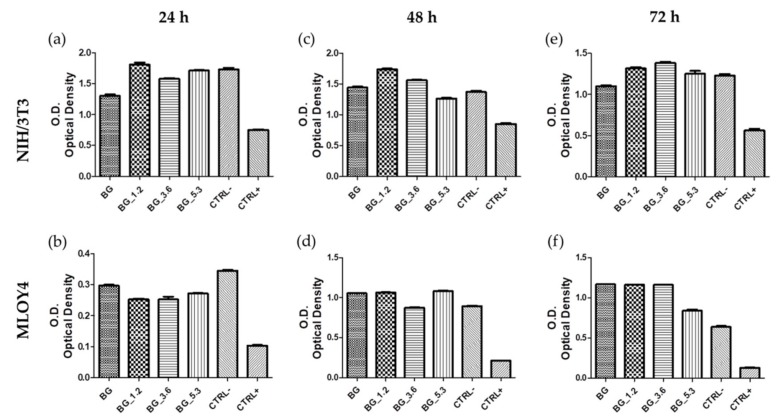
NR uptake after (**a**,**b**) 24 h, (**c**,**d**) 48 h and (**e**,**f**) 72 h for BG, BG_1.2, BG_3.6 and BG_5.3, together with negative CTRL− and positive CTRL+ controls, of (**a**,**c**,**e**) NIH/3T3 and (**b**,**d**,**f**) MLOY4 cell lines.

Cytotoxicity tests of all samples were performed using both MLO-Y4 and NIH/3T3 cell lines because of the established and recognized efficacy of the first cell line for in vitro studies involving materials for bone regeneration and foresee their interaction with osteocytes. [40] While NIH/3T3 line has been known since 1963 [41] becoming one of the most applied lines in biological studies. Results of NR uptake are shown in Figure 3, where the optical density (O.D.) obtained for material extracts are significantly higher with respect to the positive control (phenol solution) and comparable to the negative control (DMEM without extracts). Furthermore, the uptake is higher for the cerium-doped glasses with respect to BG in the case of NIH/3T3, suggesting a positive effect of cerium. This behavior is instead not detected using MLOY4, and indeed BG_5.3 caused a decrease in cell uptake at longer time (72 h).

**Figure 4:**
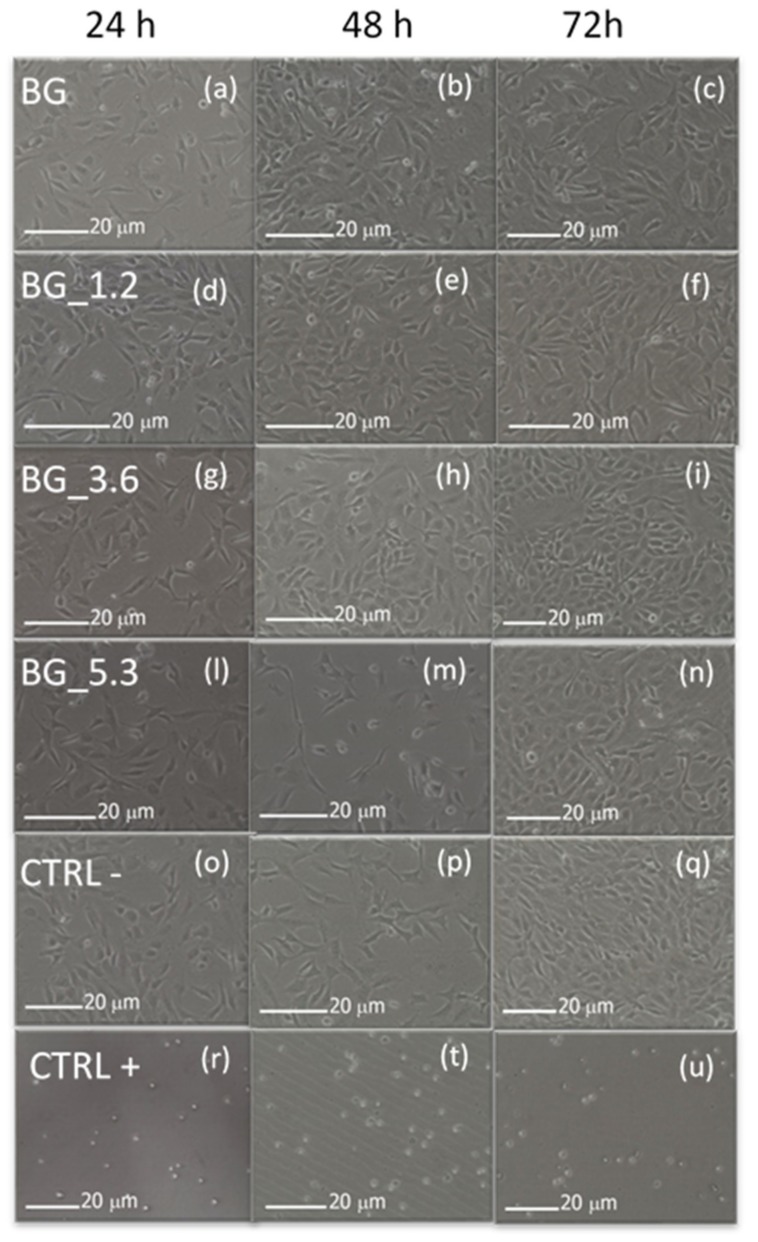
Morphological evaluation of NIH/3T3 cells after 24, 48 and 72 h in direct contact with BG, BG_1.2, BG_3.6 and BG_5.3 using optical microscopy ((**a**): BG 24 h, (**b**): BG 48 h, (**c**): BG 72 h, (**d**): BG_1.2 24 h, (**e**): BG_1.2 48 h, (**f**): BG_1.2 72 h, (**g**): BG_3.6 24 h, (**h**): BG_48 h, (**i**): BG_3.6 72 h, (**l**): BG_5.3 24 h, (**m**): BG_5.3 48 h, (**n**): BG_5.3 72 h), together with negative CTRL− ((**o**): 24 h, (**p**): 48 h, (**q**): 72 h) and positive CTRL+ controls ((**r**): 24 h, (**t**): 48 h, (**u**): 72 h).

The viability of the cultured cells was also evaluated by morphological analysis, because there is different grading of cellular reactivity. Figure 4 indicates that NIH/3T3 cells adhere onto the potential bioactive glasses and the presence of high % of CeO_2_ in the glass matrix seems not to affect the cellular adhesion on the materials. Images show grade zero (on a scale from 0 to 4) of reactivity after 24 h, i.e., no cell lysis and no reduction of cell growth. Cells appear well attached already after 24 h. At longer times (72 h) only minor aggregation and deformation were observed in the NIH/3T3 cell line, synonym of good cell viability on the biomaterials.

**Figure 5:**
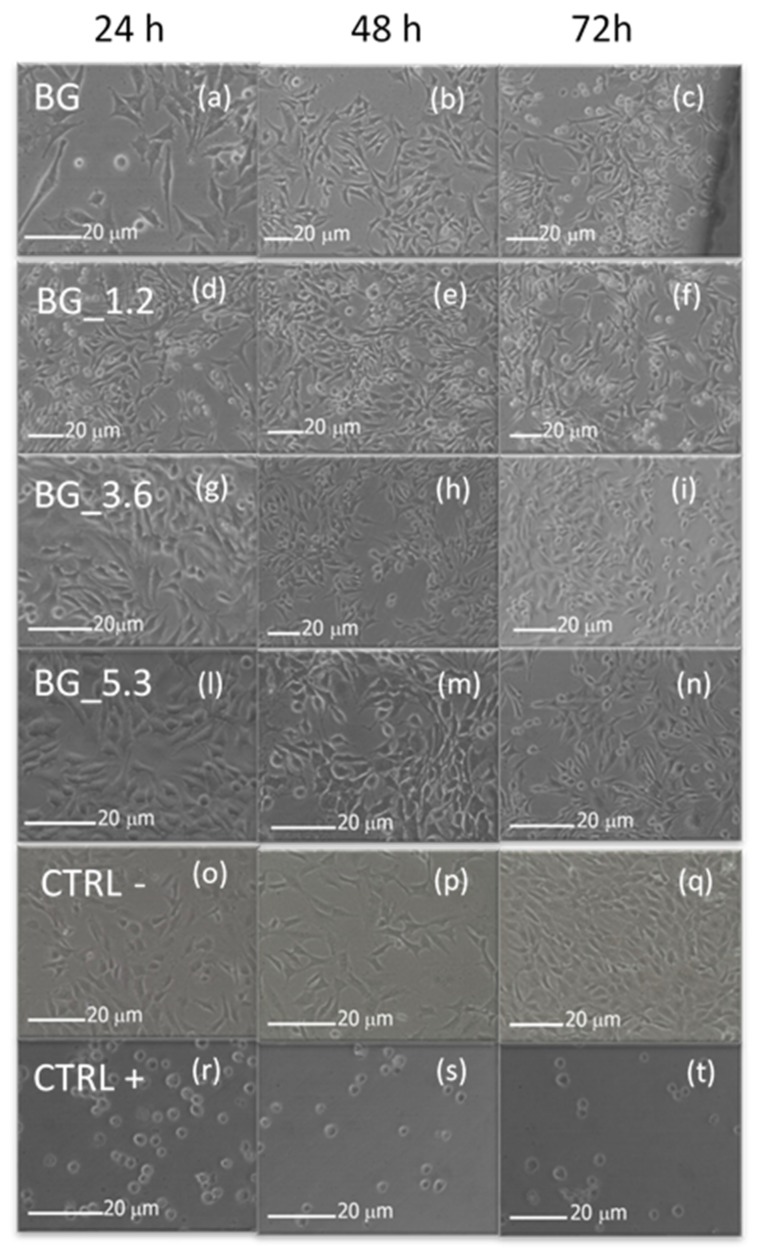
Morphological evaluation of MLOY4 cells after 24, 48 and 72 h in direct contact with BG, BG_1.2, BG_3.6 and BG_5.3 using optical microscopy ((**a**): BG 24 h, (**b**): BG 48 h, (**c**): BG 72 h, (**d**): BG_1.2 24 h, (**e**): BG_1.2 48 h, (**f**): BG_1.2 72 h, (**g**): BG_3.6 24 h, (**h**): BG_3.6 48 h, (**i**): BG_3.6 72 h, (**l**): BG_5.3 24 h, (**m**): BG_5.3 48 h, (**n**): BG_5.3 72 h), together with negative CTRL− ((**o**): 24 h, (**p**): 48 h, (**q**): 72 h) and positive CTRL+ controls ((**r**): 24 h, (**s**): 48 h, (**u**): 72 h).

Figure 5 shows MLOY4 cells with no morphological damage (no cellular lysis) in the presence of cerium-doped glasses. Cells exhibit an identical morphology to negative control (CTRL−) after 24, 48 and 72 h, with a specific increase of cellularity at the longer time.

**Figure 6:**
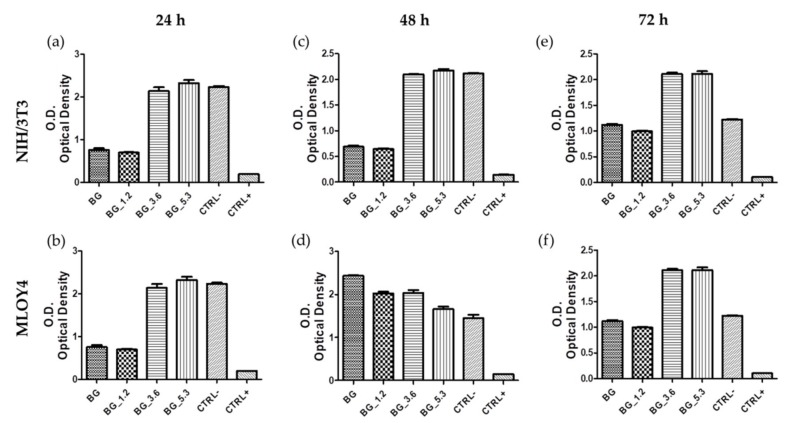
XTT tests of NIH/3T3 (left panels) and MLO-Y4 (right panels) cell lines cultured in the eluate from the glass samples after (**a**,**b**) 24, (**c**,**d**) 48 and (**e**,**f**) 72 h, together with negative CTRL− and positive CTRL+ controls.

Quantitative measurements of the cell viability were also evaluated quantitatively by means of XTT colorimetric assay (Figure 6), which has been exploited in recent years in order to evaluate the mechanisms of both cell damage and activation [36]. The glasses BG_3.6 and BG_5.3 with the highest cerium contents seem to favor the vitality of both MLO-Y4 and NIH/3T3 cells. In particular, at the longest time (72 h), these two glasses show a cellular viability superior to the negative control. These findings agree with what Genier et al. described: In fact, their results on cell viability (MTS assays) indicate that pre-treatment with nanoceria increased the cell population by 51.1% after starvation; this highlights how nanoceria seems to suppress cellular stress conditions by interfering with ROS generation [42]. This is fully in agreement with the previously-reported capacity of these glasses to mimic CAT and SOD enzymatic-activities [27].

**Figure 7:**
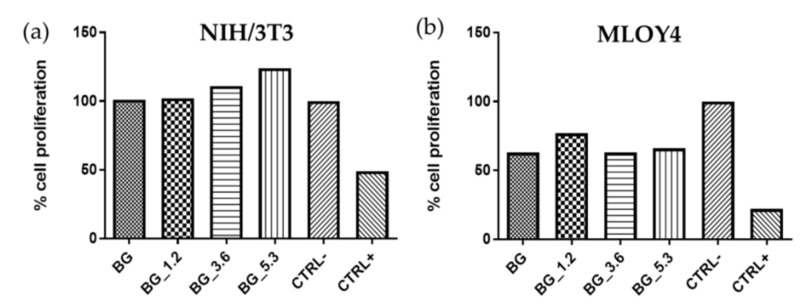
BrdU test of (**a**) NIH/3T3 and (**b**) MLO-Y4 cell lines cultured in eluate from the glass samples after 24 h, together with negative CTRL− and positive CTRL+ controls.

The proliferation of cells cultured in eluates from BG, BG_1.2, BG_3.6 and BG_5.3 was further investigated by means of BrdU assay. Such tests have been extensively used to detect in vitro and in vivo syntheses of DNA, i.e., to investigate the proliferation of cells of interest, since BrdU competes with thymidine and it can be incorporated into the DNA, thus acting as a marker, which can be tracked and detected by immunohistochemistry [37]. According to the results reported in Figure 7, the cerium-containing glasses did not negatively affect the cell proliferation. In particular, the proliferation of NIH/3T3 cells increases with increasing cerium content in the glasses, and the proliferation of cells onto BG_3.6 and BG_5.3 is higher than the negative control. From these results it is possible to suggest that the presence of cerium stimulates NIH/3T3 cell proliferation. This fact has been previously proposed: For example, low doses of cerium, at levels comparable to those found in the human serum, has a stimulatory effect on cardiac fibroblasts [43].

Naganuma and Traversa proposed that the cell proliferation and adhesion of cerium-containing materials is strictly related to the cerium oxidation state (Ce^3+^ vs. Ce^4+^) [44]. In particular, Ce^3+^ ions on the material surface inhibit cell proliferation by limiting spreading through weak cell-material interaction, while regions rich in Ce^4+^ ions promote cell proliferation. These findings seem to contrast with our previous study where the glass surface of BG_5.3 was characterize by a Ce^3+^/Ce^4+^ ratio of 3.1, showing that the glass surface is richer in Ce^3+^ than Ce^4+^ ions [25]. However, as evidenced by SEM micrograph and IR performed on the glass surfaces after DMEM soaking, the Ce^3+^ ions are mainly involved in the formation of phosphate compounds (such as CePO_4_). In fact, the morphology of neo-formed crystals on the glass surface of BG_5.3 and the Ca/P ratio trend on going from BG to BG_5.3 suggest the formation of CePO_4_ as previously reported [39].

Since the FTIR analysis supports the superficial formation of CePO_4_ but does not give certain evidences, powder X-ray diffractometric (PXRD) analyses were also conducted on glass surfaces. Unfortunately, no indication of the formation of any new crystalline phase after immersion in DMEM was obtained (due to the absence of information, data are not reported for brevity), probably due to the very low amount of the new phase formed. The formation of CePO_4_ on the glass surface is anyway in agreement with the low amount of cerium released (Table 2) strengthening the partial precipitation as cerium phosphate.

**Figure 8:**
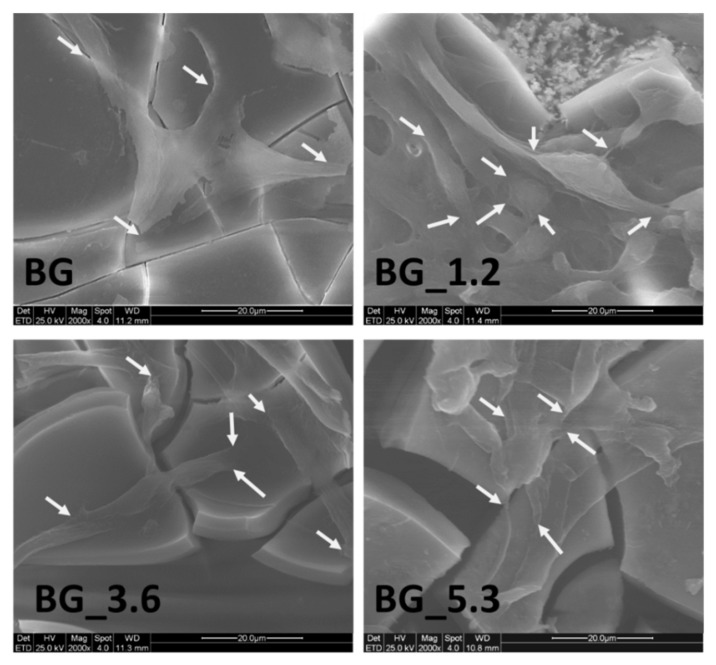
Morphology of MLO-Y4 cells incubated for 72 h on the surface of the bioactive glasses BG, BG_1.2, BG_3.6 and BG_5.3. White arrows indicate actin filaments distributed along all the cellular structure and well anchored on the surface.

All these results suggest that Ce^3+^ ions present on the surface are linked to phosphate groups, and this can explain the low ability of +3 oxidation state to inhibit the cellular proliferation. In fact, probably Ce^4+^ ions remain exposed on the glass surface, able to positively interact with proliferation cell processes as suggested by Naganume and Traversa [44]. This is confirmed by ESEM-EDS experiments performed on MLO-Y4 cells incubated for 72 h on the surface of the cerium-doped glasses (Figure 8), where cells clearly appear well attached, spread and proliferated on the surface of all the materials. Moreover, a well spread morphology with actin filaments distributed along all the cellular structure can be observed (white arrows in Figure 8).

From these results, we can confirm that the presence of high Ce^3+^ concentration on the material surface does not inhibit the cell uptake and proliferation because in our glass composition there are phosphate groups able to inhibit the Ce^3+^ activity toward the cells proliferation. In view of this, our glasses seem better materials for cell proliferation with respect to other materials containing ceria nanoparticles [44]. We have also verified that up to a content of 5.3 mol% (nominal: 5.2 mol% experimental) of CeO_2_ there are positive effects on cell proliferation. Therefore, we have expanded the compositional range investigated for CeO_2_, which was 5.0% in the current scientific literature [33].

## 4. Conclusions

This study clearly shows the ability of cerium-doped glasses based on 45S5 Bioglass® to act as potential bioactive glasses. In particular, BG_1.2 and BG_3.6 show a good bioactivity in term of HA formation during the soaking tests in DMEM. The presence on cerium ions on the material surface seems to promote cell uptake and viability, favoring also cell proliferation in the case of BG_3.6. In addition, the glass with the highest cerium content (BG_5.3) promotes cell proliferation; however, it does not show bioactivity, since the increased cerium content favors high cerium ions released in the culture medium and consequent formation of the insoluble CePO_4_ crystalline phase, which hinders the formation of HA.

On the basis of the present findings, in combination with our previous results on CAT and SOD mimetic activities [25,27], we can confidently conclude that our cerium-containing glasses BG_1.2 and BG_3.6 may be utilized in the design and development of biomaterials (e.g., scaffolds) to control cell proliferation in tissue engineering.

## Figures and Tables

**Table 1:** Nominal composition of the synthesized samples expressed as molar percentage (mol%).

Sample	SiO_2_	Na_2_O	CaO	P_2_O_5_	CeO_2_
BG	46.2	24.3	26.9	2.6	–
BG_1.2	45.6	24.0	26.6	2.6	1.2
BG_3.6	44.5	23.4	26.0	2.5	3.6
BG_5.3	43.4	23.2	25.7	2.4	5.2

**Table 2:** Si, Ca, Na, P and Ce concentrations (in ppm) after the leaching tests in DMEM solution (5% of SD). Data at *t* = 0 represent the concentrations of the elements (in ppm) before leaching tests.

Sample	72 h @ 37 °C				
	Si	Ca	Na	P	Ce
*t* = 0	0	72	3550	28	0
BG	55	77	3990	28	0.01
BG_1.2	60	78	4080	30	0.10
BG_3.6	48	74	3920	26	0.24
BG_5.3	42	73	3750	24	0.68
